# Morphological Description and Physiological Changes in the Hindgut of Female *Asiophrida xanthospilota* (Chrysomelidae, Coleoptera) Across Reproductive Stages

**DOI:** 10.3390/insects17010097

**Published:** 2026-01-14

**Authors:** Jacob M. Muinde, Ze-Qun Dong, Caren A. Ochieng, Wei Wang, Esther N. Kioko, Le Zong, Wen-Jie Li, Cong-Qiao Li, Si-Pei Liu, Zheng-Zhong Huang, Si-Qin Ge

**Affiliations:** 1 State Key Laboratory of Animal Biodiversity Conservation and Integrated Pest Management, Institute of Zoology, Chinese Academy of Sciences, Beijing 100101, China; mulwa.muinde@ioz.ac.cn (J.M.M.); ncuskdongzequn@163.com (Z.-Q.D.); caren_ochieng@mails.ucas.ac.cn (C.A.O.); wangwei@ioz.ac.cn (W.W.); zongle@ioz.ac.cn (L.Z.); liwenjie@ioz.ac.cn (W.-J.L.); licongqiao@ioz.ac.cn (C.-Q.L.); 2 University of Chinese Academy of Sciences, Beijing 100049, China; 3 National Museums of Kenya, Museum Hill, Nairobi P.O. Box 40658-00100, Kenya; ekioko@museums.or.ke

**Keywords:** hindgut, ileum, colon, rectum, rectal valve, fecal case

## Abstract

Fecal retention is a distinctive reproductive strategy in certain leaf beetles, which enables females to use accumulated fecal material to protect their eggs and enhance offspring survival. The adult flea beetle *Asiophrida xanthospilota* (Baly, 1881) is a specialist herbivore that feeds on the leaves of *Cotinus coggygria* Scop. (Anacardiaceae). Using light microscopy, scanning electron microscopy, and micro-computed tomography, we described and illustrated the hindgut anatomy of adult female *A. xanthospilota* during the pre-mated and post-mated reproductive phases. We further examined the physiological changes in the hindgut associated with fecal retention, and assessed hindgut muscle activity across these two reproductive stages. The hindgut of adult *A. xanthospilota* consists of three regions: ileum, colon, and rectum. The ileum is a thin, straight or coiled, tube enclosed by malpighian tubules and supported by circular and longitudinal muscles. The colon lies between the ileum and rectum, possesses a chitinized cuticle, and is externally covered with tracheae and tracheoles. A rectal valve separates the colon from the rectum, which forms the posterior end of the alimentary canal and is characterized by intimal spines and robust circular muscles. During the post-mated phase, fecal retention causes pronounced dilation of the hindgut, substantially increasing the volume occupied by food remnants. Electromyographic recordings revealed high hindgut muscle activity in pre-mated females, characterized by short and variable bursts, whereas post-mated females exhibited reduced activity with longer and more sustained bursts. The functional implications of these specialized structural features are discussed. Overall, these morphological and physiological adaptations enhance the fecal retention strategy by increasing fecal capacity, regulating hindgut motility, and enabling the formation of a protective fecal case around the egg mass.

## Simple Summary

The adult leaf beetle *Asiophrida xanthospilota* (Baly, 1881) is a specialist pest of *Cotinus coggygria* Scop., a widely distributed ornamental plant in northern China. This species exhibits a fecal retention strategy that protects egg masses by providing camouflage and maintaining a favorable microenvironment, thereby enhancing egg survival. Fecal retention is mediated by the hindgut, which temporarily stores ingested material prior to excretion. Using light microscopy, scanning electron microscopy, and micro-computed tomography, we investigated hindgut morphology, and associated physiological changes during the pre-mated and post-mated reproductive phases. We also assessed the functional implications of fecal retention for hindgut musculature. Our results revealed measurable increase in size in all three hindgut regions (ileum, colon, and rectum) in post-mated females. Several functionally relevant features were observed, including a chitinized inner surface of the colon, spines on the inner surface of the rectum, modifications in circular, and longitudinal muscle activity, structural changes in the rectal valve, and the presence of extensive tracheae and tracheoles. Electromyographic recordings showed high hindgut muscle activity in pre-mated beetles, whereas post-mated beetles exhibited longer contraction bursts with reduced overall activity. Overall, these findings demonstrate marked morphological and physiological differentiation of the hindgut between reproductive stages, highlighting its central role in the fecal retention strategy of *A. xanthospilota*.

## 1. Introduction

Insects display a wide range of feeding strategies that have evolved in response to dietary and environmental pressures, and these adaptations are reflected in the structural organization of the alimentary canal [1]. The alimentary canal of leaf beetles is a simple tubular structure, either straight or coiled, extending from the mouthparts to the anal region at the posterior end of the abdomen [2,3]. Generally, the role of alimentary canal includes ingestion, digestion, osmoregulation, absorption of nutrients, and excretion [4,5].

Morphologically, the alimentary canal is divided into three distinct regions: foregut, midgut, and hindgut [3,6,7,8]. The hindgut constitutes the posterior portion of the alimentarycanal, and exhibits some morphological variations in segmentation and terminology, based on the nomenclature proposed by Snodgrass (1935) [9]. Nevertheless, the hindgut in leaf beetles is commonly subdivided into the ileum, colon, and rectum. The main function of the hindgut is temporary storage of fecal material after digestion and the reabsorption of water and nutrients prior to excretion [2,10,11].

Insects undergo anatomical changes in alimentary canal especially during the metamorphosis process, and this is well documented in insects that have a dietary change during the different growth phases [12,13]. This radical transformation is notable during the pupal stage where the alimentary canal undergoes structural remodeling to suit the adult physiology [13]. During the reproductive phase, leaf beetles deposit their eggs on the leaves or twigs of host plants. However, these eggs are exposed to chemicals and natural threats such as desiccation, parasitoids and predators [14,15]. To counter these threats, some leaf beetle species have evolved protective strategies, including fecal retention, in which adult females defecate over the egg mass to form a protective fecal case [16,17]. Despite the importance of this behavior, studies investigating how leaf beetles retain substantial amounts of fecal material within the hindgut to construct these protective coverings remain limited. Furthermore, the physiological adaptations of the hindgut that facilitate fecal retention are still poorly understood.

In insects, movement is driven by muscle contractions controlled by graded electrical activity, allowing regulation of contraction speed, force, and duration [18,19,20]. Both striated and non-striated muscles exhibit graded electrical responses that produce corresponding variations in contractile responses [20]. These muscles are innervated by excitatory motor neurons classified as either fast or slow types [21]. Fast motor neurons evoke rapid, powerful contractions in response to a single action potential. Contrastingly, slow motor neurons produce more gradual contractions, with contraction amplitude regulated by neuronal firing frequency [19,22]. In the insect gut, peristalsis is driven by the rhythmic movement of the non-striated intrinsic visceral muscles, which include circular muscles that run around the gut. Their contraction produces longitudinal foldings of the epithelium, pushing the gut contents forward. Longitudinal muscles extend along the length of the gut, and their contraction, shorten the gut, facilitating wave-like peristaltic movement [20,23,24]. Hindgut motility relies on the coordinated action of these muscle layers. Therefore, behaviors that alter hindgut volume and shape, such as fecal retention, are expected to have significant physiological implications for muscle function. However, empirical evidence to support this hypothesis is limited.

The flea beetle *Asiophrida xanthospilota* (Baly, 1881) is a major specialist pest of *Cotinus coggygria* Scop. (Anacardiaceae), an ornamental plant in northern China [19,25]. Adults typically begin mating 3–5 days after eclosion, and lay eggs in clusters of 3–20 on the twigs of the host plant [26]. Females cover their eggs with a fecal case that reduces desiccation and provides protection from both biotic and abiotic threats [15,17]. This behavior suggests substantial fecal accumulation within the hindgut prior to oviposition, which may induce structural and functional modifications of the alimentary canal, particularly the hindgut. However, direct experimental evidence supporting such effects is still lacking.

Over time, studies of insect external and internal morphology have largely relied on conventional imaging techniques such as light microscopy and electron microscopy. However, these approaches have notable limitations, as they provide only two-dimensional views of structures and often require sample preparation [27]. Micro-computed tomography enables non-invasive visualization and three-dimensional reconstruction, providing comprehensive structural data from intact specimens [28,29,30]. Notably, electrophysiological techniques such as electromyography (EMG) have been widely employed to investigate the electrical activity of insect muscles, from flight muscle activation patterns to variations in muscle performance associated with different physiological states [31,32].

Using light microscopy and scanning electron microscopy, we examine the external anatomy of the hindgut from the micrographs, and noninvasive micro-computed tomography to generate three-dimensional reconstructions of its structure. We further investigate the physiological changes in the hindgut morphology between pre-mated and post-mated females. Moreover, electromyography (EMG) is used to assess and compare hindgut muscle activity across these two reproductive stages. This integrative approach provides a comprehensive analysis of the hindgut in relation to fecal retention and offers insights that may contribute to the development of new management strategies for this pest.

## 2. Materials and Methods

### 2.1. Materials

Adult male and female *A. xanthospilota* were collected from the Chinese National Botanical Garden, Beijing, China (40°00′40.23″ N, 116°12′24.02″ E) between April and October 2024. Specimens were maintained in well-aerated plastic containers and fed fresh leaves of the host plant, *Cotinus coggygria* (Scop.), under controlled laboratory conditions at the Institute of Zoology, Beijing (25 °C; 14:10 h light:dark photoperiod). Males and female beetles were reared together to allow sufficient time for mating, after which the females were transferred to separate containers and maintained on the same diet for 5–7 days until oviposition was complete. In this study, post-mated individuals refer to females that had successfully mated and were selected for experimentation only after oviposition. A control group of pre-mated female *A. xanthospilota* was reared separately and maintained under similar feeding and laboratory conditions until the experimental period.

### 2.2. Light Microscopy

10 female *A. xanthospilota* (5 pre-mated and 5 post-mated) were anesthetized at 4 °C and then dissected on a glass slide using fine forceps to remove the elytra, and ultra-fine dissection needles to remove the intersegmental membranes, muscles, and other organs that surround the hindgut. The hindgut was carefully removed, and rinsed three times in phosphate-buffered saline (PBS). The imaging was conducted using Zeiss Axio Zoom V16 stereomicroscope (PlanApo Z 1.0×/0.25 FWD 60 mm, Jena, Germany).

### 2.3. Scanning Electron Microscopy

The hindguts extracted from 10 female *A. xanthospilota* (5 pre-mated and 5 post-mated) were fixed in 2.5% glutaraldehyde for 12 h at 4 °C, and then rinsed three times in PBS for 20 min each. The samples were serially dehydrated in ethanol (75%, 80%, 85%, 90%, 95%, and twice in 100% for 30 min in each). The samples were then frozen at −80 °C for 2 h, and then dried for 1 h by a freeze dryer (Alpha 1–2 LD plus, IOZCAS, Beijing, China). The samples were mounted on a rotatable specimen holder, and sputter-coated with 33 nm gold layer for 120 s (Leica EM SCD050, IOZCAS, Beijing, China). The micrographs were observed, and captured in a scanning electron microscope at an accelerating voltage of 5–15 Kv (ESEM FEI Quanta 450, IOZCAS, Beijing China).

### 2.4. Microcomputed Tomography

4 female *A. xanthospilota* samples (2 pre-mated and 2 post-mated) were used for micro-CT scanning. The head and thorax of the samples were removed to retain the abdominal segment enclosing the hindgut. The samples were fixed in 2.5% glutaraldehyde for 12 h, and then dehydrated in a series of graded ethanol 75%, 80%, 85%, 90%, 95%, and three times in 100% (30 min in each concentration). The samples were dried in a critical point dryer (Leica EM CPD300, IOZCAS, Beijing, China), then mounted on the top of an eppendorf tube (a sample per tube). The samples were scanned in Xradia scanner (Zeiss MicroXCT-400, IOZCAS, Beijing, China) at a magnification of 4×, and images were captured at an interval of 5 s for 4.5 h. The scanned raw datasets were reconstructed using Amira software version 20.2 (Thermo Fisher Scientific, Waltham, MA, USA). The segmented structures were rendered, and visualized using the VG Studio Max 3.4.1 (Volume Graphics, Heidelberg, Germany), and final images assembled using Adobe Photoshop 21.2.1 (Adobe Inc., San Jose, CA, USA).

### 2.5. Electromyography

For examination of the electric activity of the muscles that control the contraction and relaxation of the hindgut, 8 *A. xanthospilota* samples (4 pre-mated and 4 post-mated) were dissected in a physiological saline. The use of the physiological saline was to expose the hindgut while still keeping the insect alive and preserving the exposed internal organs in physiologically viable condition. For the electromygraphic recordings, a pair of wire electrodes was carefully inserted with a 1 mm inter-electrode distance apart into the recording site at the colon region of the hindgut under stereotaxic apparatus (World Precision Instruments, Inc. PZMT/V-S, Sarasota, FL, USA), the electrodes consisted of a pair of nichrome wires (25 µm diameter, World Precision Instruments Inc., Sarasota, FL, USA) that were insulated except at their tips. The electrical signals were amplified with an AC amplifier (LabAide IX-BIO4, World Precision Instruments LLC, Sarasota, FL, USA) and filtered at high-pass (20 Hz) and low-pass (100 Hz) filters. The signals were digitized and recorded at a sampling rate of 200 samples per second using Labscribe software v4 (iWork Systems, Inc., Portsmouth, NH, USA). We examined the number of bursts, burst duration and their mean amplitude during a five second recording. The Welch two-sample *t*-test was used to compare the mean amplitude of the pre-mated and post-mated samples. Prior to analysis, normality of each group was assessed using Shapiro–Wilk test. The test statistic and associated *p*-value (*p* < 0.05) were then used to determine whether the difference in mean amplitude of pre-mated and post-mated groups was statistically significant.

### 2.6. Data Analysis

The processing of the light microscopy and scanning electron microscopy images was performed using the Adobe Photoshop version 21.2.1 (Adobe Inc., San Jose, CA, USA) and ImageJ software v1.54j (National Institutes of Health, Bethesda, MD, USA). Scanning electron microscopy results were used to assess variations in hindgut diameter and to investigate external structures involved in size changes across different reproductive stages. A schematic illustration of the gut structure based on the light microscopy was created using Adobe photoshop version 21.2.1 (Adobe Inc., San Jose, CA, USA). Electromyography data were analyzed, and visualized through Rstudio software version 4.5.1 (Posit, PBC., Boston, MA, USA), and the plates created using Adobe Photoshop version 21.2.1 (Adobe Inc., San Jose, CA, USA).

## 3. Results

### 3.1. Gross Morphology of the Hindgut

**Figure 1:**
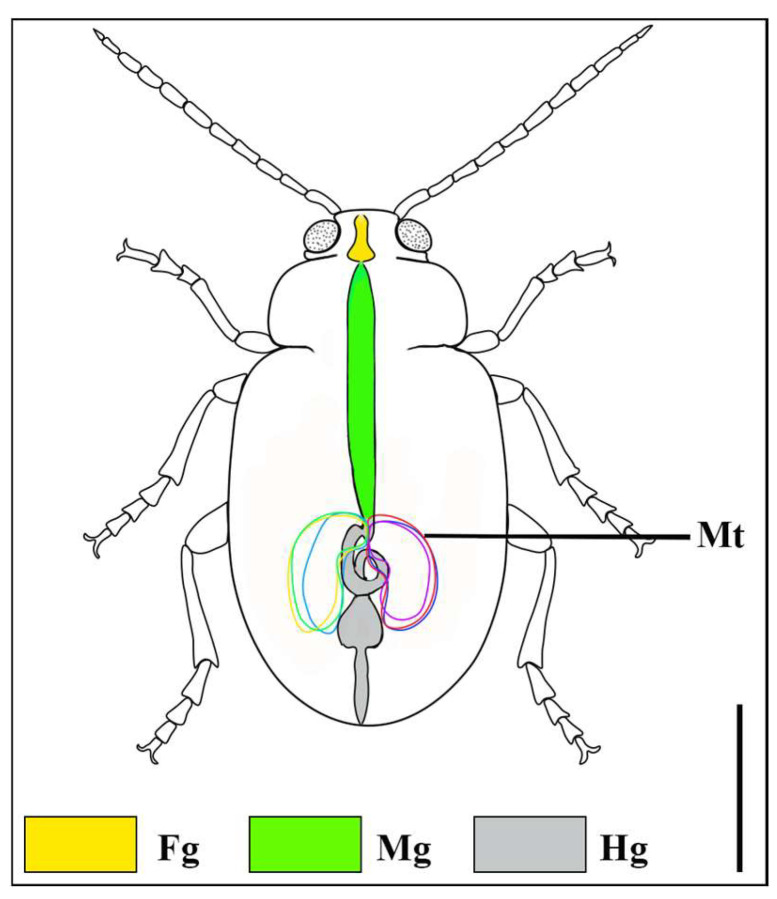
Schematic illustration of the dorsal view of *A. xanthospilota* (Chrysomelidae, Coleoptera), showing the distribution of the alimentary canal and malpighian tubules. Fg—Foregut; Mg—Midgut; Hg—Hindgut; Mt—Malpighian tubules. Scale bar = 1000 µm.

**Figure 2:**
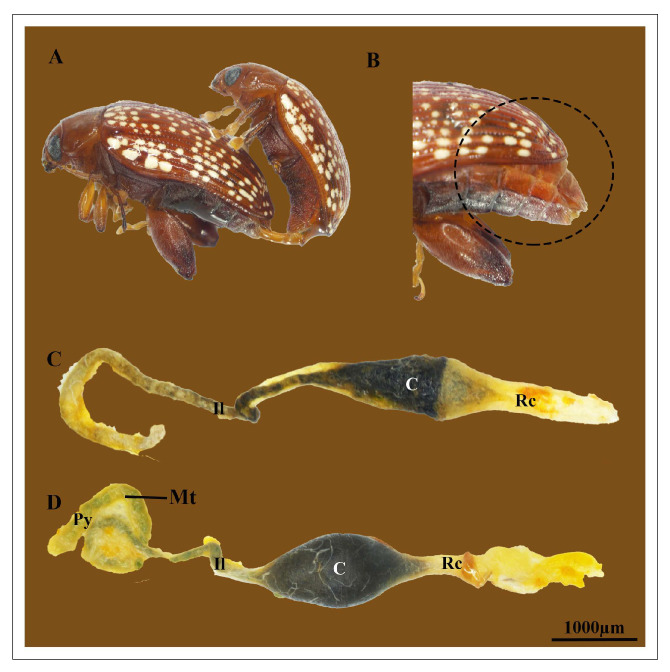
(**A**) Adult *A. xanthospilota* mating. (**B**) An external view of a protruded abdomen of the female *A. xanthospilota* due to the enlarged hindgut area. (**C**) A general view of the hindgut of a pre-mated female *A. xanthospilota* showing different regions of the hindgut. (**D**) A light microscopy image of hindgut of a post-mated female *A. xanthospilota* showing the malpighian tubules and different parts of the hindgut. Py—Pylorus; Il—Ileum; C—Colon; Rc—Rectum; Mt—Malpighian tubules.

The digestive tract of *A. xanthospilota* is divided into three major regions namely, the foregut, midgut and hindgut (Figure 1). In females, the hindgut forms a coiled structure, with the pylorus positioned at the junction between the midgut and hindgut (Figure 2C,D). Six malpighian tubules, arranged in a set of two, join the digestive tract at the pylorus (Figure 1).

**Figure 3:**
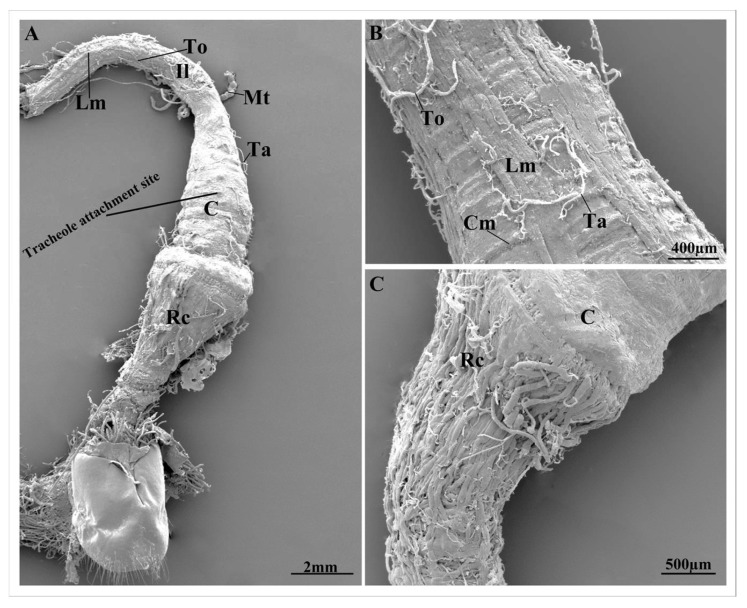
(**A**) The scanning electron microscopy micrograph of the general view of hindgut of the pre-mated female *A. xanthospilota* showing the ileum, colon, rectum, longitudinal muscles, malpighian tubule, tracheae and tracheoles. (**B**) The ileum of the pre-mated female *A. xanthospilota* showing the circular and longitudinal muscles, tracheae and tracheoles. (**C**) The distal region of the colon and rectum of the pre-mated female *A. xanthospilota*. Il—Ileum; Mt—Malpighian tubule; Ta—Tracheae; To—Tracheole; C—Colon; Rc—Rectum; Lm—Longitudinal muscles; Cm—Circular muscles.

The hindgut is composed of three structurally distinct regions: the ileum, colon, and rectum (Figure 2C,D). Ileum is the narrowest portion of the hindgut, and is surrounded by proximal malpighian tubules (Figure 2D and Figure 3A).

The ileum exhibits a coiled configuration that is surrounded by tracheae and tracheoles, and is supported by the outer poorly developed longitudinal muscles and inner thick coat of circular muscles (Figure 3A,B). In the posterior region, the ileum shows tracheolar attachment sites that appear as smooth, rounded spots on the outer surface, and extend toward the colon (Figure 3A).

**Figure 4:**
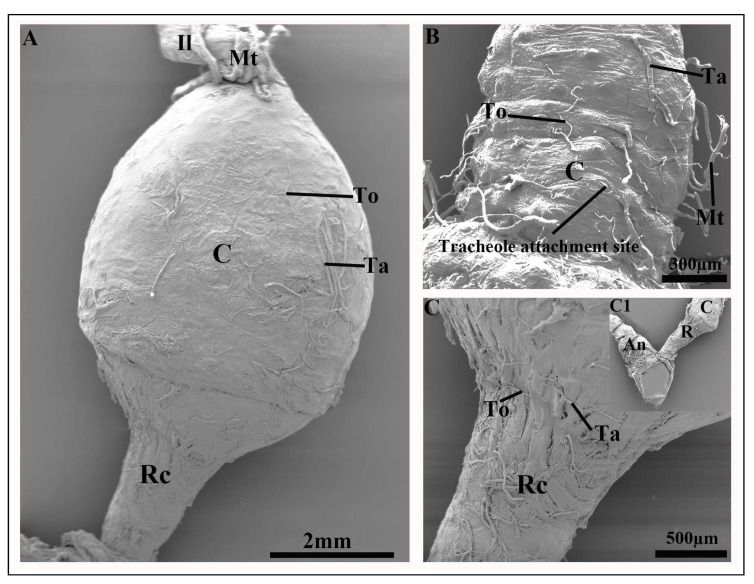
(**A**) The general view of the hindgut of post-mated female *A. xanthospilota* showing ileum, malpighian tubules, colon, rectum and trachea and tracheoles on the surface of the colon. (**B**) The ileo-colon region of post-mated female *A. xanthospilota* covered with malpighian tubules, tracheae and tracheoles. (**C**) The anterior region of the rectum of post-mated female *A. xanthospilota*. (**C1**) The distal region of the colon, rectum and the posterior region of the rectum that form the anal canal. An—Anal region; Il—Ileum; Ta—Tracheae; To—Tracheoles; Mt—Malpighian tubules; C—Colon; Rc—Rectum.

The colon is located posterior to the ileum and constitutes the widest region of the hindgut (Figure 3A and Figure 4A). Its proximal region is surrounded by the distal ends of the malpighian tubules (Figure 4A). The colon exhibits an elongated ovoid shape that is narrow at both proximal and distal ends and widest at the mid-region (Figure 4A).

**Figure 5:**
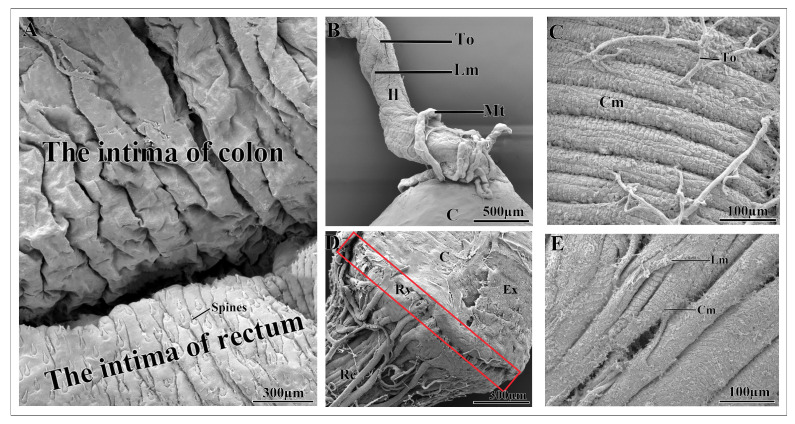
(**A**) The intima surface of the colon (upper) and rectum (lower). (**B**) The external features of the ileum surrounded by malpighian tubules. (**C**) The external surface of the colon showing the circular muscles. (**D**) The transition zone between the colon and rectum showing the rectal valve (red box). (**E**) The external surface of the rectum showing the well-developed circular and longitudinal muscles. An—Anal region; C—Colon; Rc—Rectum; Il—Ileum; To—Tracheoles; Lm—Longitudinal muscles; Mt—Malpighian tubules; Cm—Circular muscles; Rv—Rectal valve.

The colon cuticle shows a thick layer of circular muscles (Figure 5C). Externally, the colon cuticle shows numerous tracheae and tracheoles, with smooth, rounded tracheolar attachment sites that extend from the posterior region of ileum (Figure 4B). The inner surface of the colon cuticle is smooth and chitinous (Figure 5A). A distinct transition zone, the rectal valve, separates the colon from the rectum (Figure 5D).

The rectum forms a straight tubular structure at the posterior end of the colon, and is significantly narrower than the colon. It is subdivided into an anterior region adjacent to the distal end of the colon and a posterior region comprising the anal portion (Figure 3C and Figure 4A,C,C1). Externally, rectal surface is surrounded by circular muscles that can be clearly distinguished from the colon (Figure 5E). The rectum is also covered with a large number of tracheae and tracheoles (Figure 4C). The inner surface of the rectum exhibits spine-like projections (Figure 5A). The anal region constitutes the posterior region of the rectum (Figure 4C1).

**Figure 6:**
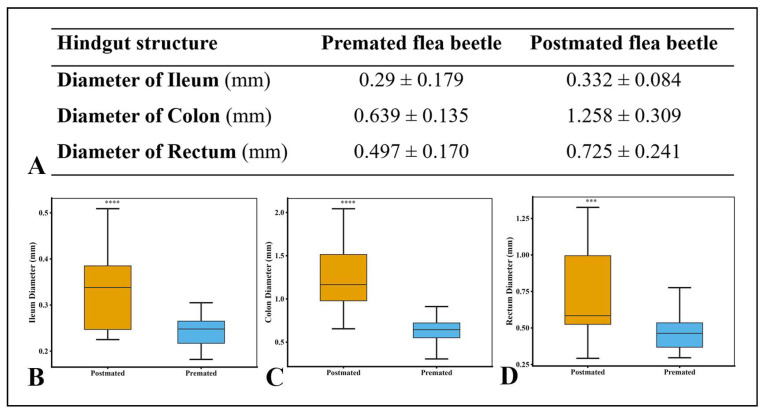
(**A**) A table showing the mean diameter of different hindgut structures in a female *A. xanthospilota*. Box plot representation of the comparison between different hindgut structures of the pre-mated and post-mated female *A. xanthospilota* (*n* = 10). (**B**) Ileum (**C**) Colon (**D**) Rectum. **** indicates a statistically significant difference between post-mated and pre-mated groups, while *** denotes a high statistically significant difference between post-mated and pre-mated groups (*p* < 0.05, Welch Two Sample *t*-test).

Clear differences were observed in the size and morphology of the hindgut between pre-mated and post-mated females. The hindgut of pre-mated females exhibited a normal appearance with no observable structural alterations. In contrast, all regions of the hindgut in post-mated females showed significant increase in size. The ileum of post-mated females was greatly distended, with a 4.2% increase in diameter relative to pre-mated individuals (Figure 4B, Figure 5B, and Figure 6A,B). Both the circular and longitudinal muscle layers were tightly stretched around the expanded ileum, and the associated malpighian tubules were visibly elongated along its entire length (Figure 5B).

The most pronounced dilation occurred in the colon, which exhibited a 61.9% increase in diameter compared with the pre-mated phase (Figure 4A, and Figure 6A,C). The rectum also showed substantial enlargement, with a 22.8% increase in diameter (Figure 6A,D). This expansion was accompanied by stretching of the external tracheolar network and marked tension in both the longitudinal and circular muscle layers (Figure 4C and Figure 5E).

### 3.2. Three-Dimensional Reconstruction

**Figure 7:**
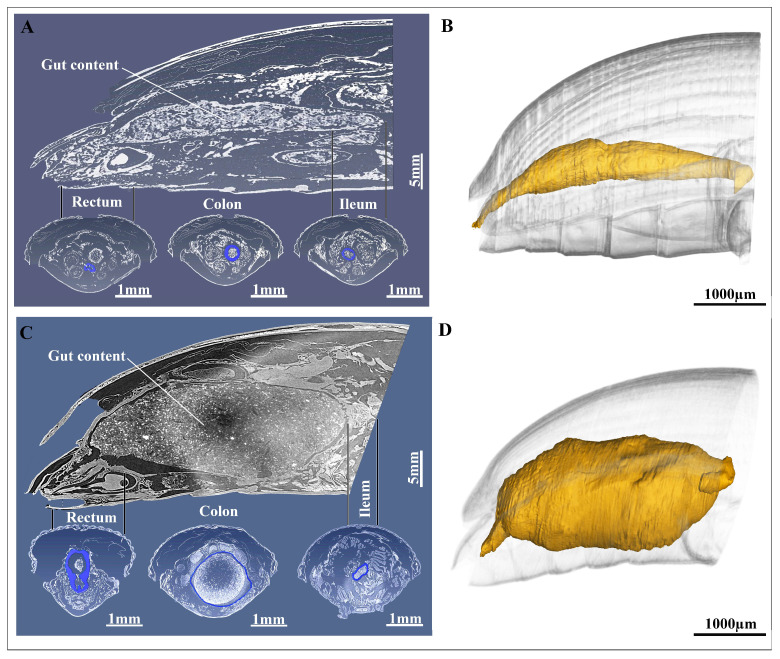
Virtual lateral 2D slice from the micro-CT data showing the three regions of the hindgut of the pre-mated female *A. xanthospilota* (**A**), and post-mated female *A. xanthospilota* (**C**). Standard-based shapes of surface reconstruction of; (**B**) the pre-mated hindgut of female *A. xanthospilota*, and (**D**) the post-mated hindgut of female *A. xanthospilota*.

After reconstruction, the three distinct regions of the hindgut, ileum, colon and rectum were easily identifiable (Figure 7A–D). The reconstruction revealed some differences in the hindgut of pre-mated and post-mated female *A. xanthospilota* hindgut (Figure 7A–D).

**Figure 8:**
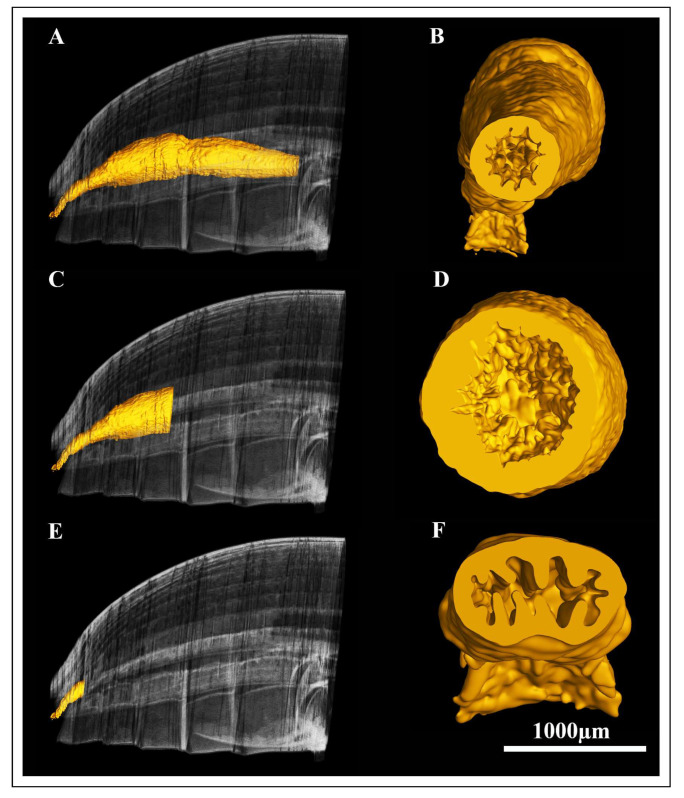
The Standard-based shapes of surface reconstruction and internal surface visualization of the hindgut regions of the pre-mated *A. xanthospilota*; (**A,B**) the ileum region, (**C,D**) the colon region and, (**E,F**) the rectum region. Scale bar = 1000 μm.

The ileum of the pre-mated females appeared narrow and elongated with a faint longitudinal ridge on the internal surface (Figure 7B and Figure 8A,B).

**Figure 9:**
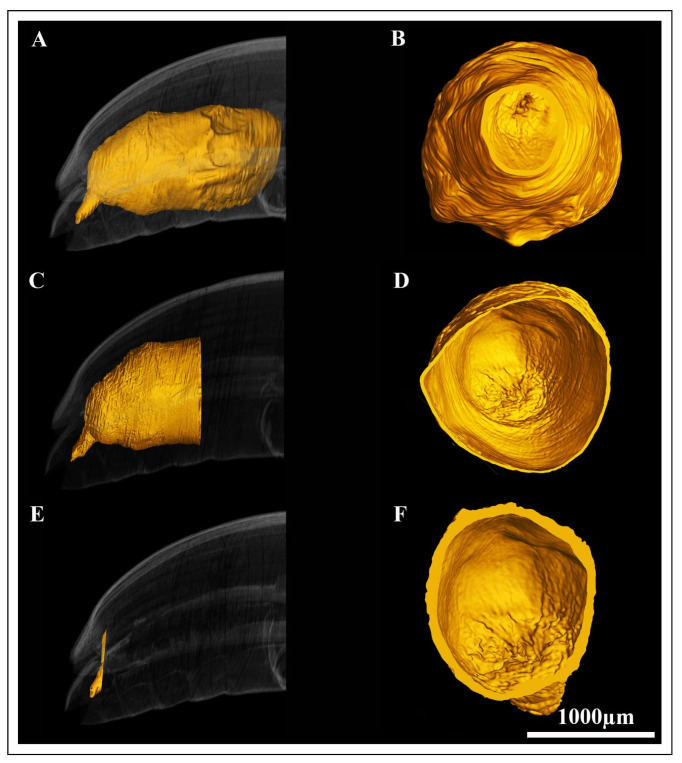
The Standard-based shapes of surface reconstruction and internal surface visualization of the hindgut regions of the post-mated *A. xanthospilota*; (**A,B**) the ileum region, (**C,D**) the colon region, and (**E,F**) the rectum region.

The foldings of the ileum decreased towards distal regions (Figure 8B). In post-mated hindgut, the lumen of ileum was enlarged and slightly irregular in shape and inner surface appeared smooth in 3D rendering (Figure 9A,B). The colon of pre-mated *A. xanthospilota* was slender and formed a gentle curvature between the narrow ileum and rectum (Figure 8C,D). The lumen of colon in pre-mated phase remained narrow and exhibited a convoluted surface (Figure 8D). The colon of post-mated phase was noticeably dilated with an expanded lumen and showed a smooth surface when visualized (Figure 9C,D). The hindgut of pre-mated phase displayed a narrow rectal lumen with the inner surface characterized by distinct foldings (Figure 8E,F). In contrast, the rectum of post-mated phase exhibited a structural remodeling where the rectal lumen was enlarged and showed less defined foldings (Figure 9E,F).

### 3.3. Muscle Electric Activity Recordings

**Figure 10:**
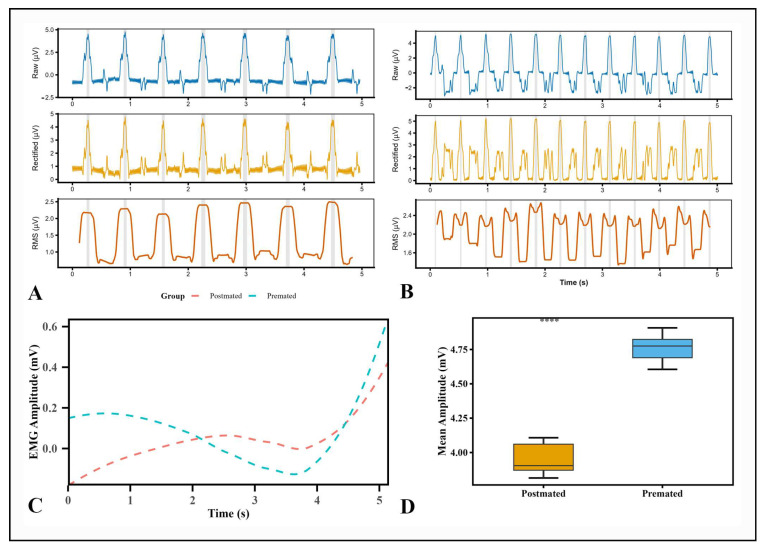
The EMG recordings of the hindgut muscles of the pre-mated female *A. xanthospilota* (**A**), and post-mated *A. xanthospilota* (**B**). Time series plots of electric activity of the hindgut muscles of the pre-mated and post-mated *A. xanthospilota* (**C**). Boxplot showing a comparison of the mean amplitude between the hindgut of the pre-mated and post-mated *A. xanthospilota* (**D**). “****” indicates a high statistically significant difference between post-mated and pre-mated groups (*p* < 0.05, Welch Two Sample *t*-test).

To measure the muscle response of the hindgut in different reproductive stages, we chose the Raw, Rectified and RMS features on the aspect of time domain for analysis (Figure 10A,B). The hindgut of pre-mated *A. xanthospilota* recorded a maximum peak excitation at 5.06µV while the hindgut of post-mated phase was 4.57 µV (Table 1).

The EMG recordings of the hindgut of pre-mated samples demonstrated shorter and more variable bursts while the hindgut of the post-mated samples showed longer and more sustained muscular activity (Figure 10A,B). The average burst duration in the pre-mated hindgut was 0.037 ± 0.007 s, whereas in the post-mated hindgut was 0.060 ± 0.009 s (Table 1). There was a significant difference in the mean amplitude between the two reproductive phases. The pre-mated hindgut exhibited a mean amplitude of 4.735 ± 0.099 µV, whereas the post-mated hindgut showed a mean amplitude of 3.956 ± 0.106 µV (Table 1). The hindgut of post-mated beetles exhibited a higher integrated amplitude 0.2574 ± 0.0406 µV than pre-mated beetles 0.18375 ± 0.0357 µV (Table 1).

## 4. Discussion

### 4.1. General Overview

The hindgut of the majority of chrysomelid beetles is divided into three regions: ileum, colon, and rectum [2,33,34]. Morphologically, the ileum, colon and rectum of the *A. xanthospilota* can be distinguished based on the surface differences. The ileum is slender, coiled, and enveloped by a well-developed circular muscle layer and longitudinal muscles. The twisted and folded organization increases the surface area for water and mineral ion reabsorption [35,36]. Similar morphological features are observed in other coleopteran species, including *Calosoma sycophanta* [34], *Capnodis tenebrionis* [37], and *Oxelytrum discicolle* [38]. The circular muscles play a crucial role in moving food remnants from the posterior end of the midgut to the hindgut. Their contractions create longitudinal foldings that help push the food content along the hindgut [20].

Externally, the ileum is also surrounded by longitudinal muscles. Although less developed than the circular muscles, these longitudinal muscles run along the ileum and generate wave-like peristaltic movements, pushing the food content to the colon [21]. The colon extends posteriorly from the ileum and is notably enlarged. Its inner surface exhibits distinct foldings of the chitinized cuticle, a characteristic feature of both foregut and hindgut regions [39,40]. This chitinous layering likely reflects variations in mechanical strength, permeability, and rigidity within the hindgut [31,41,42].

The colon is further surrounded by thick circular muscles and covered externally with tracheae and tracheoles. Similarly to the ileum, the circular and longitudinal muscles coordinate the movement of gut contents toward the rectum. The tracheal network supplies oxygen to gut cells, supporting tissue metabolism [43,44]. A rectal valve is present at the junction between the colon and rectum, and is used as a reference in differentiating between the two regions. Rectal valves have been reported in other beetle groups such as Curculionoidea [45] and Cleridae [46]. Lyal and Favreau (2015) noted that rectal valve may play an important role in absorption of water from feces and assisting in excretion [47].

The intimal surface of the rectum is thick and bears spine-like projections, which contribute mechanically and physiologically to water and ion reabsorption and the movement of fecal material toward the anal region [48,49,50]. The rectum is also supported by well-developed circular muscles that constrict the lumen, aiding in pushing the food remnants down to the anus [51,52].

### 4.2. Physiological Changes and Their Implication in Post-Mated Phase

Adult female leaf beetles have evolved multiple protective strategies to mitigate abiotic and biotic stress, with fecal retention serving as a key mechanism that shields eggs from predators, parasitoids, desiccation, and thermal extremes [11,53,54,55]. In *A. xanthospilota*, post-mated females exhibit fecal retention by covering their egg masses with fecal material, forming a protective casing that enhances egg survival by reducing water loss and deterring predation [15,56].

In this study, we observed substantial physiological modifications in the hindgut of female *A. xanthospilota* across reproductive phases. The hindgut of post-mated females was significantly enlarged, likely reflecting fecal accumulation associated with this protective behavior. These structural changes represent functional adaptations that support fecal retention during the post-mating phase, a critical process for the formation of the fecal case over the oviposited egg mass.

### 4.3. Muscle Activity Dynamics Across Reproductive Phases

The movement of food through the gut is mediated by the coordinated activity of two antagonistic muscle layers: circular and longitudinal muscles, which generate peristaltic waves [20,57,58]. However, the activity of these muscles across different reproductive phases in Coleoptera has not been previously investigated. Morphological observations indicate that the hindgut of *A. xanthospilota* undergoes physiological modifications during the post-mated phase, which in turn influence muscle performance.

Our results suggest that the enlargement of hindgut regions in post-mated females is closely associated with altered activity of the circular and longitudinal muscles. Comparative analysis between reproductive stages revealed that post-mated females exhibit longer mean burst durations. This likely reflects slower muscle contractions due to increased hindgut surface area from fecal retention. In contrast, pre-mated females showed shorter and more variable bursts. Electrophysiological recordings further indicated higher mean amplitudes in pre-mated females, consistent with stronger muscle activity and more vigorous peristaltic contractions [59]. The observed differences in muscle activity between pre-mated and post-mated hindguts may also be influenced by hormonal and neuroendocrine changes following mating; however, this hypothesis requires further experimental validation.

## 5. Conclusions

This study provides a comprehensive comparative description of the adult hindgut morphology of *A. xanthospilota*, a significant pest of *Cotinus coggygria* Scop. (Anacardiaceae). Using light microscopy, scanning electron microscopy (SEM), and micro-computed tomography (micro-CT), we identified distinct physiological changes between pre-mated and post-mated reproductive phases. Notable physiologically significant features such as variations in the intimal surfaces across hindgut regions, difference in the activity of circular and longitudinal muscles, and changes in overall hindgut dimensions were observed. Electromyographic analysis further revealed significant differences in muscle activity between reproductive stages, underscoring their role in regulating peristaltic movement.

These findings provide a foundational framework for future molecular, hormonal, and neurobiological studies on reproductive phase-dependent variations in neuromuscular activity. Future studies should incorporate basic histological techniques to enable a more detailed examination of ultrastructural changes in the hindgut, particularly on the cellular organization and cuticle deposition. Such approaches would improve understanding of cellular morphology, which is essential for interpreting functional adaptations of the hindgut in *A. xanthospilota*. In addition, quantitative analysis of fecal content across pre-mated and post-mated reproductive stages would provide valuable insight into the dynamics of fecal retention.

## Figures and Tables

**Table 1:** A table representation of different parameters examined from the electromyographic recordings of the pre-mated and post-mated reproductive phase of the *A. xanthospilota*.

	Pre-Mated Phase	Post-Mated Phase
Mean Burst Duration (s)	0.0367 ± 0.0077	0.06 ± 0.0096
Peak amplitude (µV)	5.0591 ± 0.1138	4.5681 ± 0.1287
Integrated Amplitude (µV)	0.18375 ± 0.0357	0.2574 ± 0.0406
Mean Amplitude (µV)	4.7347 ± 0.0956	3.956 ± 0.106

## Data Availability

The original contributions presented in this study are included in the article. However, the raw micro-CT data can be made available upon request to the corresponding author.
